# An investigative framework to facilitate epidemiological thinking during herd problem-solving

**DOI:** 10.1186/s13620-017-0089-6

**Published:** 2017-04-19

**Authors:** Simon J. More, Michael L. Doherty, Luke O’Grady

**Affiliations:** UCD School of Veterinary Medicine, University College Dublin, Belfield, Dublin, 4 Ireland

**Keywords:** Diagnostic approaches, Herd problem-solving, Epidemiology, Investigation, Education

## Abstract

Veterinary clinicians and students commonly use diagnostic approaches appropriate for individual cases when conducting herd problem-solving. However, these approaches can be problematic, in part because they make limited use of epidemiological principles and methods, which has clear application during the investigation of herd problems. In this paper, we provide an overview of diagnostic approaches that are used when investigating individual animal cases, and the challenges faced when these approaches are directly translated from the individual to the herd. Further, we propose an investigative framework to facilitate epidemiological thinking during herd problem-solving.

A number of different approaches are used when making a diagnosis on an individual animal, including pattern recognition, hypothetico-deductive reasoning, and the key abnormality method. Methods commonly applied to individuals are often adapted for herd problem-solving: ‘comparison with best practice’ being a herd-level adaptation of pattern recognition, and ‘differential diagnoses’ a herd-level adaptation of hypothetico-deductive reasoning. These approaches can be effective, however, challenges can arise. Herds are complex; a collection of individual cows, but also additional layers relating to environment, management, feeding etc. It is unrealistic to expect seamless translation of diagnostic approaches from the individual to the herd. Comparison with best practice is time-consuming and prioritisation of actions can be problematic, whereas differential diagnoses can lead to ‘pathogen hunting’, particularly in complex cases.

Epidemiology is the science of understanding disease in populations. The focus is on the population, underpinned by principles and utilising methods that seek to allow us to generate solid conclusions from apparently uncontrolled situations. In this paper, we argue for the inclusion of epidemiological principles and methods as an additional tool for herd problem-solving, and outline an investigative framework, with examples, to effectively incorporate these principles and methods with other diagnostic approaches during herd problem-solving. Relevant measures of performance are identified, and measures of case frequencies are calculated and compared across time, in space and among animal groupings, to identify patterns, clues and plausible hypotheses, consistent with potential biological processes. With this knowledge, the subsequent investigation (relevant on-farm activities, diagnostic testing and other examinations) can be focused, and actions prioritised (specifically, those actions that are likely to make the greatest difference in addressing the problem if enacted).

In our experience, this investigative framework is an effective teaching tool, facilitating epidemiological thinking among students during herd problem-solving. It is a generic and robust process, suited to many herd-based problems.

## Background

Clinical case management encompasses a well-defined course that includes data collection (history, signalment, physical examination, paraclinical tests), diagnosis and the development of a management plan. These activities are underpinned by clinical decision-making, which allows clinicians to modify their approach in response to each individual clinical situation [1].

In this paper, we focus on ‘making a diagnosis’, which is a central tenet of clinical veterinary medicine. It allows veterinarians to recognise a disease, and to assign this disease label to a case that presents with particular clinical or pathological characteristics. Ultimately, assigning a diagnosis facilitates short and long-term case management, including the selection of appropriate and effective therapeutic actions, and allows for ease of communication regarding prognosis with clients and colleagues.

A number of different diagnostic approaches can be used, including pattern recognition and hypothetico-deductive reasoning [1, 2], which will be considered later. In an educational sense, relevant to Bloom’s taxonomy of educational objectives [3], the diagnostic approach requires a deep understanding of relevant issues; that is, an understanding of facts, but also of concepts, processes, procedures and principles. In the veterinary literature, emphasis has been placed on the challenges of uncertainty that are faced by veterinary clinicians. It is recognised, for example, that veterinary clinicians generally face greater uncertainty and weaker levels of evidence than their human counterparts [2]. A review is available of explicit quantitative methods to assist with clinical decision-making in the face of uncertainty, including decision analysis and clinical diagnostic decision support systems [2]. Further, much has been written on the clinical use and interpretation of diagnostic tests, both with respect to individuals and populations [4], and on the concept of evidence-based veterinary medicine (EBVM) [5], where clinical decisions are based on the explicit use of best-available evidence in addition to clinical expertise. Critical appraisal of available literature is central to EBVM, using robust methods such as systematic reviews and meta-analyses [6], including recent examples both from companion [7] and production [8, 9] animals.

Herd problem-solving relies on the application of diagnostic methods to animal populations. Many aspects of this process are well described, in particular the application of epidemiological principles and methods to herd problem-solving, particularly in a Northern American setting [10–13]. However, there are elements that are currently unclear. Firstly, there has been limited discussion on diagnostic approaches in populations. It is uncertain, for example, whether pattern recognition and hypothetico-deductive reasoning can each be effectively applied in such settings. Secondly, numeric methods are particularly useful in large populations, such as larger feedlots, but less well suited to more modest herd sizes of say 50–100 cows, which is more the European norm. Finally, in the authors’ experience, many clinicians are uncertain how these methods can be practically and efficiently incorporated into everyday practice.

In our experience at University College Dublin (UCD), veterinary clinicians and students commonly use diagnostic approaches appropriate for individual cases when conducting herd problem-solving. However, direct translation of these approaches is problematic, in part because they make limited use of epidemiological principles and methods, which has clear application during the investigation of herd problems.

In this paper, we provide an overview of diagnostic approaches that are used when investigating individual animal cases, and the challenges faced when these approaches are directly translated from the individual to the herd. Further, we propose an investigative framework to facilitate epidemiological thinking during herd problem-solving.

## Investigating individual cases

Making a diagnosis at the level of the individual bovine animal may be relatively straightforward, such as in the case of a fracture of the metatarsus, or more complex and challenging such as recumbency in a calf or cattle presenting with neurological signs.

Radostits et al. describe a number of different approaches that are used by veterinarians when making a diagnosis on an individual animal, including the three outlined below, namely pattern recognition, hypothetico-deductive reasoning, and the key abnormality method [1]. These approaches to individual cases are not exclusive, and are often used unconsciously, in combination or interchangeably, depending on the clinical circumstances and prior experience of the practicing veterinarian.

*Pattern recognition*, that is, recognition of the disease syndrome, after comparison with previous cases. This approach is common among experienced clinicians.

*Hypothetico-deductive reasoning.* Initially, multiple plausible diagnostic hypotheses (differential diagnoses) are generated, based on presenting clues. Then, the clinician asks questions and conducts clinical examinations, to test (support or discount) each hypothesis. The process of hypothesis and deduction continues until one hypothesis is preferred over all others.

The *key abnormality method* requires that clinicians rely on their knowledge of normal structure and function to identify and evaluate the key abnormality or clinical cue. It is particularly valuable during the investigation of difficult cases. At the level of the individual animal, Radostits et al. describe a five-step process, including sequential determination of:

- The abnormality of function present,
- The system or body as a whole or organ affected,
- The location of the lesion within the affected system or organ,
- The type of lesion, and
- The specific cause of the lesion [1].

The *key abnormality method* is used extensively by staff and students in the UCD School of Veterinary Medicine at University College Dublin. It is underpinned by a deep understanding of structure and function relevant to the case in hand, with a focus on the *what?* and the *how?*, to understand the *why?* The diagnostic process is informed by the clinical examination, which is a thorough and meticulous standard operating procedure of data collection and processing, including signalment/disease history and a physical examination of the animal. With these data, diagnostic hypotheses are developed, informed by an understanding of the clinical presentation in terms of structure and function, and by a body of knowledge of common diseases. This information guides the judicious selection of laboratory tests and other diagnostic aids (radiography, ultrasound etc.).

Using this approach, students are equipped to deal with both simple and complex cases using a process that emphasises attention to detail and a calm, methodical and thorough approach. Data from history and clinical examination are often more powerful than laboratory data, and most errors are due to a lack of thoroughness rather than a lack of knowledge. Importantly, students are neither clutching for diagnoses nor unjustifiably wed to one. Further, injudicious use of diagnostic testing and polypharmy can each be avoided.

An outline of this approach, using a recumbent calf as an example, is presented in Table 1.

**Table 1:** Use of the key abnormality method to make a diagnosis when presented with a recumbent calf

1. Understand structure and function relevant to recumbency in a calf The nervous system • Central nervous system (CNS) (cerebral cortex/cerebellum) • Peripheral nervous system (peripheral nerve damage) • Femoral nerve (noting that most will stand with assistance) The musculoskeletal system • Muscle weakness (dehydration/metabolic acidosis/anoxia/malnutrition) • Muscle pathology (myopathy) • Skeleton (fractures/pain)
2. Ascertain the history/signalment Take a detailed history based on the signalment of the animal in question and directed towards determining whether the most common causes of disease Take a generic history followed by key, case-specific questions. • Key information includes age, signalment, the timeline of clinical signs and the progression of disease • Key, case-specific questions • Has the calf been recumbent since birth? • Was the calf able to stand at birth but subsequently became recumbent? • Was there dystocia? • What is the herd bovine viral diarrhoea (BVD) status?
3. Perform a general clinical examination Determine which body system(s) are primarily involved by: • Establishing the most likely location of any lesions • Establishing the type of lesions Key systems of interest in the recumbent calf are: • The central and peripheral nervous system • The musculoskeletal system General clinical examination • Is the calf dull, depressed? • Is the calf bright, alert and reactive? • Can the calf stand once it is assisted to rise? • What is the hydration status? • Is there a suck reflex? • Mucous membranes? • Hypopyon present? If central and peripheral nervous system is implicated continue to full neurological examination. If musculoskeletal system is implicated continue to full musculoskeletal examination
4. Consider and rank differential diagnoses Rank differential diagnoses in order of probability of occurrence. Apply clinical knowledge, experience and rational thinking. Common diseases should be at the top of your list of differential diagnoses. * a. Calf recumbent since birth* i. Calf is dull and/or depressed • History of severe dystocia, absence of suck reflex • Most probable diagnosis: *Post-dystocia cerebral anoxia with metabolic acidosis* *(*less common diagnoses: *hydrocephalus, umbilical haemorrhage)* ii. Calf is bright, alert and reactive • History of severe dystocia (assess lower and upper motor neuron function to help localise spinal cord segment involved) • Most probable diagnosis: *Spinal cord trauma* • History of severe dystocia, musculoskeletal lesions • Most probable diagnosis: *Fracture of femur, metacarpal, first phalanx (P1)* • History of severe dystocia, genuflexion, laxity of patella and neurogenic atrophy of the quadriceps femoris • Most probable diagnosis: *Femoral nerve paralysis* (particularly if bilateral) • Intention tremor (head bobbing), if able to stand a wide-based stance • Most probable diagnosis: *Cerebellar hypoplasia* (BVD) • Obvious pelvic asymmetry when standing • Most probable diagnosis: *Hip dislocation* *(*less common diagnoses: *hip dislocation, vitamin E/selenium myopathy)* * b. Calf became recumbent approximately 10 days after birth* i. Calf is dull and/or depressed • Diarrhoea and varying degrees of metabolic acidosis (assess hydration) • Most probable diagnosis: Enterotoxigenic *Escherichia coli* (ETEC)/*rotavirus diarrhoea* • Petechiation and/or hypopyon • Most probable diagnosis*: Septicaemic colibacillosis* *(*less common diagnosis: *metabolic acidosis in the absence of diarrhoea)* ii. Calf is bright, alert and reactive • CNS implicated following neurological examination • Most probable diagnosis: *Spinal abscess* *(*less common diagnosis: *congenital heart defect)*

## Direct translation to herd problem-solving

As with individual animal medicine, ‘making a diagnosis’ is also a central tenet of herd health investigations. A correct herd-level diagnosis is important and generally needed, before proceeding to recommendations for appropriate herd-level strategies for control and prevention.

It is common, and understandable, that clinicians use familiar diagnostic approaches during herd health visits; essentially, the same diagnostic method for herds as they would normally do for individuals. The following approaches to diagnosis of herd-level problems are adaptations of methods commonly applied to individuals:

- *Comparison with best-practice.* Using this approach, the investigation includes a detailed review of farm practices relevant to this case (a farm audit, relevant to the presenting problem), with the clinician (perhaps subconsciously) comparing practices on the affected farm with what they would view as best-practice, social norms or published guidelines. Table 2 presents data relevant to a herd mastitis investigation. This can be thought of as a herd-level adaptation of ‘*pattern recognition*’, where the diagnosis is based on a perceived or identified gap or deficiency in comparison to what would be expected on ‘good’ farms. As one example, during the investigation of a herd mastitis problem, a veterinarian may determine that the volume of teat dip or spray applied post-milking is less than optimal (generally at least 10 or 15 mL of solution is recommended during post-milking dipping or spraying, respectively, to achieve complete coverage of the teats) [14]. Remedying these deficiencies often forms the basis of action for improvement, for example, ensuring that all steps of the ten point plan for mastitis control are in place.

**Table 2:** Data relevant to a herd mastitis investigation. From Dairy Australia [17, 18]

Farm profile	
Milk cultures	
Individual cow cell count analysis	
Milking machine dry test	
Performance tests of milking machines	
Milking routines	
Clinical cases	
Teat condition	
Cow behaviour, milking time per cow	
Completeness of milking, cluster alignment	
Teat disinfection	
The environment
- *Differential diagnoses,* being a herd-level adaptation of ‘*hypothetico-deductive reasoning*’. Using this approach, a series of plausible differential diagnoses are generated, typically based on initial questioning, clinical examination of problem animals to give herd-level clues, to then perform hypothetico-deductive reasoning. Often this requires the use of additional diagnostic testing to identify the single most-likely hypothesis, e.g. investigating a problem of calf diarrhoea to identify the causal pathogen. This approach is then used to employ known efficacious controls relevant to the diagnosis.

These two diagnostic approaches may either be used in isolation or in conjunction and often prove effective in investigating herd problems. In many situations, however, particular challenges may arise.

*Comparison with best practice.* There are several potential issues:

- This approach can be very time-consuming, as it relies on a comprehensive understanding of all farm activities relevant to the presenting problem. Table 2 illustrates this point, highlighting key issues to be considered during a herd mastitis investigation.
- Using this approach, a clinician is (subconsciously) comparing observed with best-practice. However, best-practice will vary between clinicians, influenced by many factors including clinical experience. Logically, therefore, lists for improvement (based on perceived differences between the affected farm and best-practice) will also vary between clinicians.
- Farmer opinions of best practice may differ from those of the clinician leading to conflict when communicating recommendations.
- Using this approach in isolation, it is challenging to prioritise the list for action, i.e. to identify those actions that, if addressed, would make the greatest difference with respect to the problem under investigation. This is especially true if the list is extensive, and can often lead to ‘fix everything’ recommendations.

*Differential diagnoses*. This approach is of greatest use in simple cases, with a single disease process. However, an evaluation of differential diagnoses is of limited assistance in complex cases, e.g. those with multiple overlapping disease processes [1] or where multiple pathogens, some possibly incidental, are identified during diagnostic testing. Using this diagnostic approach, there is a danger that ‘pathogen hunting’ might dominate the herd-level investigation. Again, where multiple pathogens are present, the challenge of prioritisation arises. In this instance, availability and cost of treatments (curative or preventive) may strongly influence the decision making process, i.e. “treat what’s treatable” recommendations.

## Incorporating epidemiological thinking into herd problem-solving

### Overview

In comparison to the individual, the herd is a much more complex place. Certainly a herd is a collection of individual cows, however, this is ‘overlaid’ with additional layers relating to environment, management, feeding etc. There is a complex interaction of disease with many factors, including nutritional strategy and the housing environment. Further, the influence of social and attitudinal factors is substantial. It is understandable, therefore, that herd investigations can be very challenging. Given this background, it does seem unrealistic to expect seamless translation of diagnostic approaches from the individual to the herd. Indeed, the difficulties outlined above, when using *‘comparison with best practice’* or *‘differential diagnoses’* approaches for herd problem-solving, can be directly traced to the complexities of reality at farm level.

When considering diagnostic approaches to individual cases, we have previously highlighted the importance of a deep understanding of structure and function relevant to the case in hand. At the individual level, the focus is on the *what?* and the *how?*, to understand the *why?* At the herd level, it is important that the focus is underpinned by a deep understanding of both the individual and the broader population. Further, we need to consider incorporating additional tools to enable us to make sense of this complexity.

In the traditional disciplines with which we are most familiar, such as physiology, microbiology or pathology, the focus is at the level of the individual, the organ or the cell, and our reasoning is underpinned by a clear understanding of processes of normality or disease. As highlighted previously, the key questions being asked are *‘what?’* and *‘how?’*, to help us to better understand the ‘*why?*’ In contrast, with epidemiology the focus is on the population, underpinned by principles and utilising methods that seek to allow us to generate solid conclusions from apparently uncontrolled situations. Here, the key questions are ‘*whether?*’, *‘when?’*, ‘*where?*’, *‘which?* and *‘how much?’*, providing perspectives on the ‘*why?*’ As such, epidemiology is a science that is well suited to herd investigations. In short, epidemiological principles and methods should be incorporated into diagnostic approaches to herd problem-solving, complementing traditional disciplines.

### Epidemiological principles

There are a number of epidemiological principles relevant to herd problem-solving, which we list below. This list is not exhaustive and we refer interested readers to introductory texts about epidemiological principles and methods [15, 16]. These principles are a valuable addition to herd problem-solving, regardless of the quantitative skills of the investigating clinician. They provide a useful guide to the act and science of ‘epidemiological thinking’.

i. *Cause, control, prevention*
  In a herd investigation, we generally focus on one or more of the following three issues:
*Cause* – factors or determinants that influence health and disease (a determinant when altered would produce a change in the frequency or characteristics of disease) [15]. Key questions for consideration include: What factor(s) are contributing to the observed effect? Are there multiple causes (a multifactorial problem)? Is there a causal pathway, with several contributing factors? Which points along the causal pathway are most influential in creating the problem?
*Control* – efforts directed towards reducing the frequency or severity of existing disease to levels that can be justified biologically and/or economically [15]. Key questions for consideration include: What can be done to stop it? What potential actions can be taken to effectively break the causal pathway?
*Prevention* - measures to prevent exposure to causal factors, including the exclusion of infectious agents from a farm (for example, through bioexclusion) or to protect a given population in an infected area (for example, through vaccination) [15]. Key questions for consideration include: What can be done to prevent it happening again? What actions can either remove causal factors or reduce the frequency or impact of their occurrence?

The issue of cause is particularly important in a herd investigation, noting that there is rarely a single cause; rather our interest will generally focus on causal pathways, on drivers and on risk factors. Herd problem-solving is typically conducted where performance is substantially less than the farm goals that were set.

ii. *Patterns, clues and hypotheses*
  Epidemiology is a science with a particular interest in patterns. In common with all scientific disciplines, diseases (or other conditions) are not considered random event, rather there is one or more reasons for their emergence. Given this, epidemiologists seek an understanding of patterns of presentation in a population, as n follows:
  In time (our key related questions are *when does the condition present?*, *whether the condition presents during the time period(s) of interest?*, *how many animals [number, percentage of total] are affected during the time period(s) of interest?*)
  In space (our key related questions are *where does the condition present?, whether the condition presents in the location(s) of interest?*, *how many animals [number, percentage of total] are affected in the location(s) of interest?*)
  Among different animal groupings (age, sex, management groups etc.) (our key related questions are *which animal groupings are affected?*, *whether the condition presents in the animal grouping(s) of interest?*, *how many animals [number, percentage of total] in the grouping(s) of interest are affected?*)

These patterns are used as clues, helping to identify hypotheses of potential causation that plausibly fit with the patterns observed. Further investigation is then needed to critically evaluate each of these hypotheses.

iii. *Performance and activity*
  In an epidemiological context, it is critical to distinguish performance and activity:
*Performance* refers to the output, or what is achieved, and is generally measured using key performance indicators (KPIs). With respect to milk quality, for example, this could relate to the bulk milk somatic cell count achieved during a defined period of time
  In contrast, *activity* refers to processes, or what was done. Again with respect to milk quality, this could include in-parlour practices such stripping, teat dipping and cow segregation, the blanket use of dry cow therapy, the regular servicing of the milking machine etc.

### Incorporating epidemiological thinking into herd problem-solving

In this section, we outline an investigative framework to effectively incorporate epidemiological principles and methods with other diagnostic approaches during herd problem-solving. We acknowledge that many – perhaps all – aspects of this framework may be familiar to veterinarians, given the importance of applied epidemiology to clinical practice. The approach is outlined in Table 3, with an example, but is generic and should be adaptable regardless of the clinical setting. The enhanced approach mirrors the optimal approach at the level of the individual animal, where clinicians draw on a deep understanding of structure and function, and use key clinical clues to generate focusing questions and plausible hypotheses. Using these optimal diagnostic approaches, both for the individual case and for herd problem-solving, it should be possible for the busy clinician to focus their efforts as quickly as possible during the investigation.

**Table 3:** An epidemiological approach to herd problem-solving, using mastitis as an example

1. Initially focusing on performance [Individual milk recording data are generally needed] a. Building the framework i. Defining the problem, in terms of performance An increase in somatic cell count (SCC) ii. Developing a case definition For example: • A cow with SCC >200,000 cells/mL at the current milk recording, or • A cow with SCC>200,000 cells/mL at three or more tests during the current lactation iii. Calculating simple measures of case frequency and of epidemiological association • Case frequency • by parity • by stage of lactation • Epidemiological association • Odds ratio, relative risk b. Looking for patterns • In time • In space • Among different animal groupings c. Critically evaluating patterns and clues • Generate plausible hypotheses, given: • The patterns observed, and • A sound understanding of relevant biological processes (such as the source and spread of infectious agents, see Figure 1)	
2. Then focusing on relevant farm activities [Conducted through on-farm investigation, observation, interview etc.] A focused investigation of relevant farm activities, consistent with all plausible hypotheses. This could include some or all of the following: i. The milking parlour The milking machine • Dry test • Capacity • Vacuum, airflows • Pulsators • Liners and other rubberware • Performance testing The milking routine • Prior to cups-on • Teat cleanliness • Let-down • During milking • Milking time per cow • Cow behaviour, milking time per cow, overmilking • Completeness of milking, cluster alignment • Teat cup slips • After cups-off • Teat condition • Teat disinfection Detection of clinical cases ii. The environment Around calving During housing At pasture • The walkways • The grazing areas	
3. Conducting focused diagnostic testing and other examinations [Focusing on the problem animals] • Milk cultures	
4. Developing recommendations, and communicating these to farmers • Facilitating understanding • With the farmer • Prioritising actions • Developing recommendations	

#### Step 1: Initially focusing on performance

**Fig. 1:**
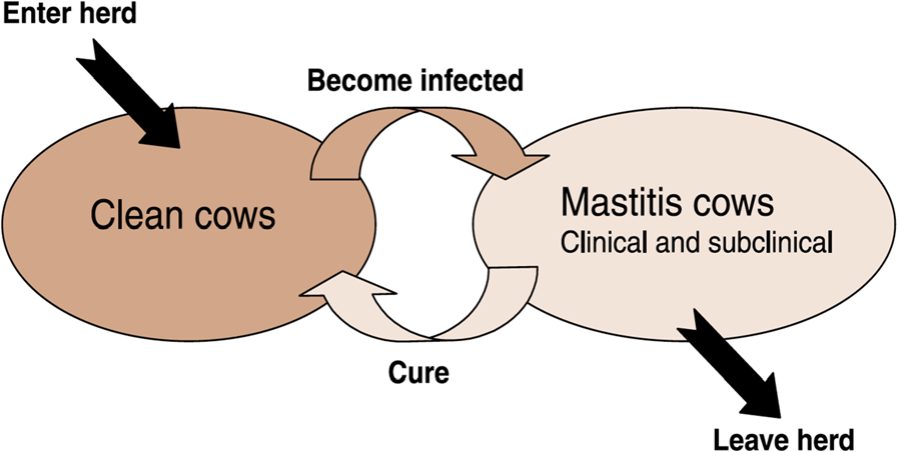
A herd mastitis dynamics chart [17], which which can be used to underpin a sound understanding of source and spread with respect to infectious mastitis. Dairy Australia, used with permission

- Building the foundations
  During this initial stage of the investigation, it is critical to focus on *performance*, rather than *activity.* Indeed, any thoughts or concerns regarding activities on the farm should initially be ignored.
  Firstly, the problem needs to be framed in terms of performance, for example, ‘an increase in calf mortality’, ‘an increase in somatic cell count (SCC)’, or ‘an increase in the incidence of lameness’.
  Then, a case definition is developed to enable cases to be identified and counted (for example, ‘a calf that died during the last month’, ‘a cow with at least one SCC reading greater in 200,000 cells/mL during the current lactation’, ‘a cow with a lameness score of 3 or greater’). The case definition is very important, as it enables the investigator to count cases, and to distinguish cases from non-case (or control) animals. A numeric focus such as this is not a surprise, noting that epidemiology is a science underpinned by numbers.
  These data allow a clinician to then calculate simple measures of case frequency and of epidemiological association. At its simplest, the case frequency will include the number of cases (the numerator) and the total number of animals at risk (generally the number of cases and non-cases, the denominator). Initially, an overall figure would be calculated for all eligible animals in the herd. Subsequently, the problem could be investigated by considering the epidemiological dimensions of time, space and different animal groupings (age, sex, management groups etc.), as relevant. For example, during a milk quality investigation, the % of cows with an SCC >200,000 cells/mL might be calculated during each month of the current lactation (the case frequency in time). Similarly, the % of cows with an SCC >200,000 cells/mL will be calculated by age (among lactation 1, among lactation 2, etc.) (the case frequency among animal groupings).
- Looking for patterns
  With this information, case frequencies are then compared to determine whether patterns (systematic differences between cases and controls) may be present, either in time, in space and between different animal groups. Formal statistical evaluation could be considered in larger herds (where sample size is sufficient), however, this is often not feasible, and visual comparison will suffice.
- Identifying clues
  Epidemiological thinking relies on the sequential concepts of patterns, clues and hypotheses. If patterns are present (for example, ‘a seasonal pattern of presentation is apparent’, or ‘there appears to be an increased risk among first lactation animals as compared with older cows’), these can be considered as clues, contributing to the development of plausible hypotheses with respect to potential causation. During this process of hypothesis generation, it is critical that decision-making is underpinned by a sound understanding of potential biological processes. For example, Fig. 1 describes the concepts of source and spread of infectious agents with respect to bovine mastitis in a dairy herd (Fig. 1). Therefore, a comprehensive list of plausible hypotheses are developed, consistent with both the underpinning biological processes and the epidemiological patterns of presentation.

#### Step 2: Then considering relevant farm activities

During step 2, the clinician only considers those activities that have the potential to plausibly contribute to any of the hypotheses identified in step 1. In other words, step 1 should be of considerable assistance to the clinician, as it allows the potential for considerable focus during the latter stages of the on-farm investigation. This is in contrast to the *‘comparison with best-practice’* method mentioned previously, where all aspects of herd activities would need to be scrutinised. During this step, a process of *hypothetico-deductive reasoning* is used, as outlined previously. Guided by the plausible hypotheses that were developed during step 1, the clinician identifies relevant farm activities and seeks further information about each, through questions and by other means.

#### Step 3: Focused diagnostic testing and other examinations

Further investigation is generally needed to critically evaluate each of the plausible hypotheses that were previously generated. Specifically, clinical examinations and sample collection may be conducted to test (support or discount) each of these hypotheses. This is generally restricted to animals that meet the case definition, however, other animals could also be considered if biologically relevant. For example, early cases or controls may each be important during the investigation of outbreaks of respiratory disease or diarrhoea, to clarify the status and/or contribution of apparently health animals. The process of hypothesis and deduction continues until one hypothesis is preferred over all others.

#### Step 4: Developing recommendations, and communicating these to farmers

When using either the *‘comparison with best practice’* and *‘differential diagnoses’* herd diagnostic approaches, there is a limited ability to prioritise in the face of complexity. This prevents decision-making based on the ranking of likely casual factors and the efficacy or efficiency of interventions. In addition, there is a danger that recommendations will be ‘cherry-picked’ by the farmer, leading to frustration on the part of the clinician when key recommendations are not followed. In contrast, these epidemiological approaches can also assist in prioritising actions, specifically those actions that are likely to make the greatest difference in addressing the problem if enacted.

Ultimately, it is critical for clinicians to be seen to add value beyond generic disease control practices. With individual animal cases, the treatments and solutions are generally under the influence of the clinician. In contrast, the resolution of most herd issues resides with the actions of the farmer. Therefore, communication and facilitating behavioural change are critical attributes of effective herd clinicians. The role of the clinician in the herd scenario should be to facilitate understanding, to present potential solutions based on tackling the causal factor(s), and to help to prioritise actions.

## Conclusions

The investigative framework, with the addition of epidemiological principles and methods to existing, well-established diagnostic approaches, is used extensively by UCD farm animal clinicians. We have found it to be an effective teaching tool, facilitating epidemiological thinking among students during herd problem-solving. Further, we have found it to be a generic and robust process, suited to many herd-based problems. The framework is underpinned by a sound understanding of potential biological processes by the clinician, somewhat similar to the deep understanding of structure and function relevant to the case in hand, when investigating individual cases using the key abnormality approach. The incorporation of epidemiological principles and methods is well justifiable during herd investigations given the need to address complexities inherent in a herd-level focus.
